# Translational Research Studies Unraveling the Origins of Psoriatic Arthritis: Moving Beyond Skin and Joints

**DOI:** 10.3389/fmed.2021.711823

**Published:** 2021-08-17

**Authors:** Janne W. Bolt, Chaja M. J. van Ansenwoude, Ihsan Hammoura, Marleen G. van de Sande, Lisa G. M. van Baarsen

**Affiliations:** ^1^Department of Rheumatology & Clinical Immunology, Amsterdam Institute for Infection & Immunity, Amsterdam UMC, University of Amsterdam, Amsterdam, Netherlands; ^2^Department of Experimental Immunology, Amsterdam Institute for Infection & Immunity, Amsterdam UMC, University of Amsterdam, Amsterdam, Netherlands; ^3^Amsterdam Rheumatology & Immunology Center (ARC), Academic Medical Center, Amsterdam, Netherlands

**Keywords:** early psoriatic arthritis, psoriasis, immunopathogenesis, translational, animal models

## Abstract

Patients with psoriatic arthritis (PsA) are suffering from a decreased quality of life despite currently available treatments. In the latest years, novel therapies targeting the IL-17/IL-23 and TNF pathways improved clinical outcome. Despite this, remission of disease is not achieved in a considerable group of patients, continuous treatment is very often required to reach clinical remission, and prevention of PsA in patients with psoriasis (PsO) is currently impossible. A better understanding of PsA pathogenesis is required to develop novel treatment strategies that target inflammation and destruction more effectively and at an early stage of the disease, or even before clinically manifest disease. The skin is considered as one of the sites of onset of immune activation, triggering the inflammatory cascade in PsA. PsO develops into PsA in 30% of the PsO patients. Influenced by environmental and genetic factors, the inflammatory process in the skin, entheses, and/or gut may evolve into synovial tissue inflammation, characterized by influx of immune cells. The exact role of the innate and adaptive immune cells in disease pathogenesis is not completely known. The involvement of activated IL-17A+ T cells could implicate early immunomodulatory events generated in lymphoid organs thereby shaping the pathogenic inflammatory response leading to disease. In this perspective article, we provide the reader with an overview of the current literature regarding the immunological changes observed during the earliest stages of PsA. Moreover, we will postulate future areas of translational research aimed at increasing our knowledge on the molecular mechanisms driving disease development, which will aid the identification of novel potential therapeutic targets to limit the progression of PsA.

## Key Messages

- Most translational research studies investigated blood, skin, and synovial tissues from PsA patients with established disease and showed that IL-23/IL-17 axis and TNF are of importance for the pathogenesis of PsA together with DAMPs, DCs, PMNs, keratinocytes and T cells.
- Consensus is needed to define the different phases of PsA development starting from the preclinical phase.
- Prospective studies that follow PsO patients toward the development of PsA are required to identify predictive biomarkers as well as the molecular and cellular processes associated with development of PsA.
- Translational research studies in tissues beyond skin and synovium, such as lymphoid organs, will shed new light on the immunological processes potentially initiating disease pathogenesis.
- To test potential targets for treatment, improved animal models are needed that better resemble the transition from PsO to PsA.

## Introduction

Psoriatic arthritis (PsA) is a heterogeneous immune-mediated inflammatory disease with musculoskeletal symptoms including arthritis, enthesitis, dactylitis, and axial involvement. Psoriasis (PsO), affecting both skin and nails, is present in most PsA patients (1). Other non-musculoskeletal features linked to PsA are uveitis and colitis, which are observed in a smaller number of patients. Prevalence of PsA is equal in women and men, who suffer from subsequent structural damage and loss of quality of life (2, 3). In 80% of the patients PsA is preceded by PsO and 30% of the PsO patients develop PsA over an average time of 10 years (2–8). Recognition and characterization of this subgroup of PsO patients with an increased risk for (developing) PsA could facilitate early identification of PsA and for this reason screening tools for dermatologists to ease the recognition of PsA have been developed (5, 9–11). These screening tools are used with different levels of success (5), and more than one out of ten PsA diagnoses is missed in the PsO population (12–14). Diagnostic delays until 5 years are reported (15) and this hampers the start of treatment in the early phase of disease. Diagnosis in the first year after the onset of symptoms has been shown to decrease structural damage and improve clinical and patient-reported outcomes (16–19). These improvements are probably caused by the earlier initiation of treatment. Despite revolutionary improvements in treatment over the last years, disease remission is only achieved in up to 15% of the patients (20), and it is not possible to prevent the transition from PsO to PsA. Preventive treatment strategies are hampered by the absence of predictive biomarkers for PsA development that allow the identification of those patients with a very high risk to develop disease, and lack of knowledge on potential cellular or molecular treatment targets. Many efforts have been made to delineate the molecular pathways involved in PsA pathogenesis. However, knowledge on altered molecular pathways in individuals with an increased risk of developing PsA is lacking. Insight into such pathways may lead to the discovery of novel drug targets for preventive treatment in those at-risk individuals and may lead to the identification of biomarkers associated with PsA development. Here we will first give an overview of the current understanding of PsA from both a clinical and immunological perspective based on studies in animal models and humans focused on the earliest, even preclinical, phases of PsA. Secondly, we will describe future areas of translational research, which will increase our knowledge of the molecular mechanisms driving disease development.

## Challenges in Early Recognition of Psoriatic Arthritis

Clinical recognition of PsA in an early phase of the disease and consensus on its terminology is needed to identify and study early molecular changes in PsA patients. Recognition of PsA is however challenging due to the subtlety of the first symptoms and as a result, around 15% of all PsA patients are missed at the dermatology clinics (5, 12). Various clinical screening tools have been developed to accelerate early identification (9, 10, 21). These tools are based on studies that evaluated risk factors for having PsA when having PsO and contain various clinical features (22), such as nail (23) and scalp involvement, increased severity of PsO, family history of PsO (23), a high body mass index and late onset of PsO (6, 24–27), to be more frequently present in these patients. However, the downside of these questionnaire-based tools is the varying specificities and sensitivities, especially when applied in populations with a lower prevalence of PsA (9, 10, 21). The concept and terminology of early PsA have only recently gained popularity among researchers in the field of PsA. The challenges that occur in the recognition and terminology of early PsA hamper the availability of a clear overview of literature focusing on this topic. Currently, collaborating initiatives between dermatologists and rheumatologists are focusing on early PsA and these initiatives will hopefully accomplish more consensus on duration and terminology in early PsA (28). The importance of adopting such a concept has been proven in rheumatoid arthritis (RA), where consensus on early disease stages resulted in more uniform terminology for early disease and facilitated clinical trials in patients with early, or even at-risk, RA. There is no consensus on the exact terminology and duration of the early phase of PsA (28). In this perspective article, we will refer to early PsA as the first 2 years after diagnosis of PsA although others have suggested a shorter period after PsA diagnosis to be defined as early disease (22).

## Immunopathogenesis of Psoriatic Arthritis: Lessons Learned From Fundamental Research

**Figure 1:**
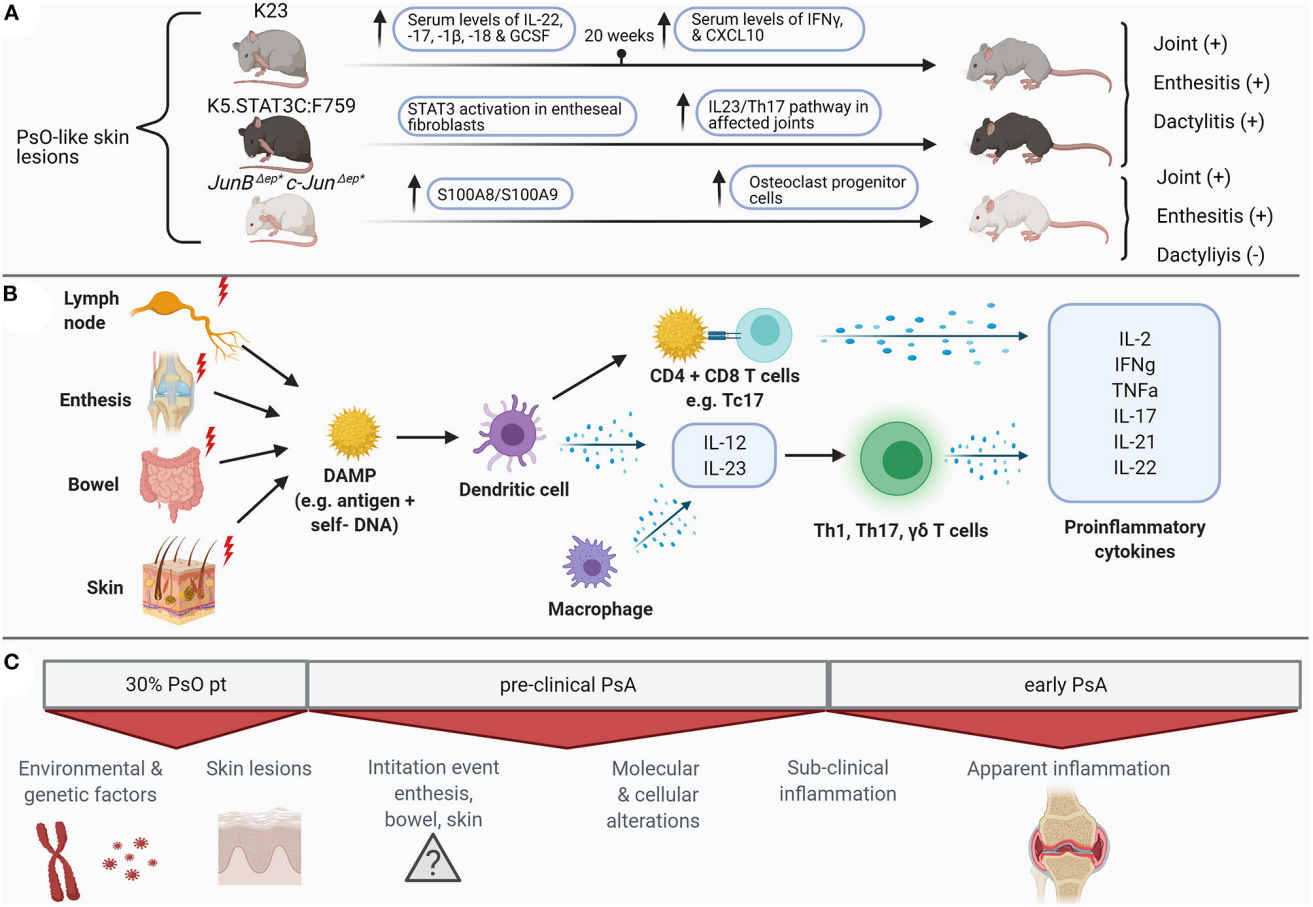
Schematic overview of preclinical and clinical molecular **(B)** and clinical alterations **(C)** in psoriatic arthritis in humans and the featured mice models **(A)** for psoriatic arthritis. PsO, psoriasis; IL, interleukin; IFN, interferon; GCSF, granulocyte colony-stimulating factor; CXCL, chemokine ligand; STAT, signal transducer and activator of transcription; Th, T helper cells; DAMP, danger associated molecular patterns; CD, cluster of differentiation; Tc, cytotoxic T cells; TNF, tumor necrosis factor; PsA, psoriatic arthritis. Figure created with BioRender.com.

The clear challenges in recognizing early PsA are reflected by the limited number of human studies that have focused on elucidating the immunological drivers of early PsA. For this reason, current knowledge regarding immunopathogenesis is mostly derived from human samples obtained from patients with established PsA (summarized in Figure 1). Here we will discuss the current knowledge on immunopathogenesis in established and early PsA and highlight some of the pivotal cytokines involved in PsA pathogenesis. Moreover, we will compare human studies with several animal models reflecting clinical and immunological aspects of human PsA. The current available animal models, shown in Table 1, include different mechanisms for disease induction. These models reflect at least partly human PsA pathogenesis, but none of these models are completely representative.

**Table 1:** Characteristics of animals models for psoriatic arthritis and their contribution to understanding disease pathogenesis.

**Animal model**	**Mice**	**Type of induction**	**Clinical or imaging disease manifestation**	**Histological disease manifestation**	**Suggested contribution to understanding pathogenesis PsA**
R26STAT3Cstopfl/fl CD4Cre transgenic mice (29, 30)	R26STAT3Cstopfl/fl CD4Cre mice	Hyperactive expression of STAT3C in naïve T cells, resulting in expansion of Th17 cells.	Psoriasis-like skin inflammation (+) Arthritis (–) Enthesitis (–) Dactylitis (–) Bone erosion (+) Osteopenia (+)	Psoriasis-like skin inflammation (+) Synovitis (+) Enthesitis (+)	STAT3 signaling and Th17 cells are of importance for PsA-like disease development. RANKL expression and subsequent osteoclast differentiation is related to IL-17 from Th17 cells that act on mesenchymal cells.
K5.Stat3C:F759 transgenic mice (31)	K5.Stat3C:F759 mice	Hyperactive expression of STAT3C in keratinocytes and constitutive activation of IL-6 signaling that together lead to persistent STAT3 activation due to impaired SOCS3-negative feedback with induction of IL17A.	Psoriasis-like skin inflammation (+) Arthritis (+) Enthesitis (+) Dactylitis (+)	Psoriasis-like skin inflammation (+) Synovitis (+)	Facilitating role of skin inflammation for joint inflammation via crosstalk between keratinocytes and entheseal fibroblasts mediated by IL-6 and IL-23/Th17-associated STAT3 activation.
Mannan induced psoriatic disease in B10Q mice (32, 33)	B10Q mice	Intra peritoneal injection of Mannan, a ligand for mannose receptors at the host-fungus interactions and known as the major trigger of interleukin-17 (IL-17) production in B10Q mice.	Psoriasis-like skin inflammation (+) Arthritis^*^ (+) Enthesitis (–) Dactylitis (–) New bone formation (+)	Psoriasis-like skin inflammation (+) Synovitis (–) Enthesitis (+) New bone formation (+)	Supporting role for macrophages in inducing PsA-like disease via triggering of IL-17A secretion by γδT cells and ILC3. These studies showed that reactive oxygen species (ROS) production by macrophages is protective for PsA-like disease whereas nitric oxygen species promote disease.
Mannan induced psoriatic disease in *B10Q.Ncf1*^m1j/m1^ mice (32, 33)	*B10Q.Ncf1*^m1j/m1^ mice	The Ncf1 mutation impairs superoxide anion production by the NOX2 complex and impaired ROS production by macrophages. This worsened the clinical phenotype.	Psoriasis-like skin inflammation (+) Arthritis (+) Enthesitis (+) New bone formation (+)	Psoriasis-like skin inflammation (+) Synovitis (+) Enthesitis (+) New bone formation (+) Erosions (+)	
JunB/c-Jun double-mutant mice (34)	JunBf/f c-Junf/f K5-Cre-ERT mice	Inducible epidermal deletion of JunB and c-Jun. In humans the JunB transcription factor is localized in psoriasis susceptibility locus 6 and regulates cell proliferation, differentiation, stress responses and cytokine expression.	Psoriasis-like skin inflammation (+) Arthritis (+) Dactylitis (–)	Psoriasis-like skin inflammation (+) Synovitis (+) Bone erosions (+)	Epidermal modulation of JunB leads to the secretion of chemokines and cytokines that recruit inflammatory cells in the skin resulting in PsO-like skin lesions and later development of arthritis. This underscores that epidermal changes can induce joint inflammation.
K23 mice (35)	R23/K14CreERT2 mice	Inducible-conditional IL-23 overexpression in keratinocytes.	Psoriasis-like skin inflammation (+) Arthritis (+) Enthesitis (–) Dactylitis (+)	Psoriasis-like skin inflammation (+) Synovitis (+) Enthesitis (+) Dactylitis (+) Bone erosions (+)	IL-23 plays a role in the initiation PsO, which later transits into PsA.
IL-17A minicircle gene transfer model in C57BL/6J mice (36)	C57BL/6J mice	Systemic overexpression of mouse IL-17A *in vivo* through injection of IL-17A minicircle construct in tail vein resulting in transduction of hepatocytes.	Psoriasis-like skin inflammation (+) Arthritis (–) Enthesitis (–) Dactylitis (–) Bone erosion (+)	Psoriasis-like skin inflammation (+) Bone erosions (+)	Link between IL-17, myelopoeiesis and neutrophils. IL-17A induces PsO like skin lesions. IL-17 can induce bone erosions in absence of synovitis.

*PsA, psoriatic arthritis; STAT, signal transducer and activator of transcription; Th, T helper cells; RANKL, receptor activator of nuclear factor kappa-β ligand; IL, interleukin; SOCS3, Suppressor of cytokine signaling 3; ILC, innate lymphoid cells; NOX, nicotinamide adenine dinucleotide phosphate oxidase; ROS, reactive oxygen species; NO, nitrogen oxide; PsO, psoriasis*.

* *Animals showed joint swelling without synovitis on histology*.

### Initiation of Psoriatic Arthritis

It has been postulated that the initiation of PsA occurs after triggering by environmental factors in gut, entheses, or skin in patients who are genetically more susceptible to development of this disease (37). The genetics of PsA have been reviewed in detail (38–41). In short, both HLA and non-HLA coding genes are associated with PsA. HLA coding genes linked to PsA have supported the role for peptide binding and T cell activation in PsA pathogenesis (40). In the non-HLA coding groups genes are related to innate immunity, cytokines (TNF, IFN, and IL-23/17) and antigen processing and presentation (38). However, genes that are specifically associated with development of PsA in PsO patients remain scarce (39). Two HLA-B27 haplotypes (C^*^01 and C^*^02, respectively) (42, 43) and IL13 gene polymorphism were suggested to be independent associated with PsA development in PsO patients (44, 45). The role of the gut microbiome in PsA pathogenesis has recently gained interest (46). Compared to healthy individuals, PsO and PsA patients have different gut microbiome profiles (46). PsO and PsA patients have overlapping gut microbiome profiles, but differences between the two are also observed (46). Next to microbiome alterations, higher levels of fecal calprotectin and asymptomatic gastrointestinal inflammation are observed in PsA patients (47). It is suggested that intestinal permeability, alterations in immune homeostasis in the bowel and an imbalance of fatty-acid-producing bacteria could be involved in the immunopathogenesis of PsA, but these topics will need further investigation to understand their exact involvement in PsA (46). It is suggested that enthesitis can trigger arthritis via the so-called synovio-entheseal complex (SEC) which highlights the regional relationship between enthesis and neighboring synovial membrane (48). Tissue studies investigating molecular alterations in human entheses that could drive PsA onset are highly challenging to perform and thus scarce (49). The few available studies in SpA have shown early vascularization and immune cell infiltration in entheses (50), and have implicated a possible disease-inducing role via entheseal mesenchymal stromal cells (51) and the production of pivotal pro-inflammatory cytokines by resident myeloid cells (52). In contrast to the gut and the entheses, several studies have investigated the underlying molecular alterations in synovium of PsA patients and psoriatic skin lesions. Of interest is the comparison between skin of PsO and PsA patients, which is informative to understand the relation between the two diseases. These kinds of studies are unfortunately scarce (53, 54), but cross-sectional comparisons do point to molecular differences between skin of PsO and PsA patients (54).

### From Psoriasis to Psoriatic Arthritis

In psoriatic skin, an imbalance in the composition of the microbiome (55) and colonization with pathogens (56) is observed, which is suggested to play a role in the external triggering of disease. These findings, as well as mechanical trauma, are thought to initiate the onset of psoriasis by activating keratinocytes (57). Activated and stressed keratinocytes and infiltrating neutrophils then release self-DNA and antimicrobial peptides (AMPs) (57) of which some are overlapping in PsO and PsA (58). The complex of self-DNA and AMP is protected from extracellular nuclease degradation and functions as a danger-associated molecular pattern (DAMP) in the psoriatic skin where it activates abundantly present plasmacytoid dendritic cells (pDCs) and myeloid dendritic cells (mDCs) (59–61). The disruption of these complexes via topical Imiquimod treatment, which is a toll like receptor ligand, was shown to result in the alleviation of the psoriatic skin lesions in the imiquimod-induced psoriasis-like mice model (62) and supports the importance of these complexes in PsA. After the activation by DAMPS, the dermal mDCs probably migrate to lymph nodes (LN) and stimulate differentiation and proliferation of T cells through the production of cytokines such as IL-12 and IL-23 resulting in Th1 and Th17 differentiation (63–65). The exact location and mechanisms of T cell activation have not yet been uncovered (66), but it is proposed that in skin and synovium this takes place through a common antigen as analyses revealed the presence of similar T cell clones in both tissues of PsA patients (67). This theory is supported by the observation of the antigen LL37 (a cationic AMP) being present in both synovial tissue and skin (60, 68). In one study, even antibodies targeting this antigen have been detected in synovial fluid and plasma of patients (60), but the exact role of these autoantibodies in PsA pathogenesis needs further investigation and confirmation. Upon activation, T cells migrate to peripheral tissues such as joints and skin, which is reflected by the abundant amount of Th17 cells in peripheral blood and synovial fluid already in the early stages of PsA (69). This finding suggests that the upregulation of Th1 and Th17 cells and their effector cytokines are essential for the start of a continuous inflammatory response (69). Recently, IL-17A+ CD8+ T cells gained interest, also due to their strong association with the major histocompatibility complex (MHC) class I, of which the genes predispose to PsA (70). CD8+ T cells are polyfunctional enabling the production of a wide range of cytokines both in skin and joints (66). In blood of PsA patients, more memory CD8+ T cells have been found compared to healthy controls and PsO patients (53), underlining a possible important role of memory CD8+ T cells in PsA. Also in synovial fluid of PsA patients, these CD8+ cells are abundantly present and are clonally expanded more extensively than CD4+ T cells (66, 71). Transcriptome analysis showed that these expanded CD8+ T cells in the joint are memory cells expressing tissue-homing as well as tissue-resident markers (Trm cells) (71). The role of T cells in steering PsA development is supported by a study in JunB/c-Jun double-mutant mice, in which mice keratinocyte-specific JunB and c-Jun transcription factors are deleted in adult mice (by inducible knock out) causing psoriatic-like plaques with accumulation of neutrophils, macrophages, and T cells in the and joint inflammation (34). It was shown that when inducing absence of functional B and T cells by creating Rag2-deficient JunB/c-Jun double-mutant mice (34), these mice develop psoriatic skin lesions (34) though with a strong reduction in inflammation of the joints. It is not clear which exact mechanisms are responsible for the migration of T cells to different peripheral tissues. It is hypothesized that in PsA T cells migrate from the skin or peripheral blood to the other affected tissues (72). In 2 mice studies specific molecular alterations in the skin resulted in joint inflammation with a clear role for T cells in the JunB/C-Jun double mutant mice (29, 31, 34) (Table 1). However detailed migration of T cells was not investigated. The final inflammatory response in PsA is characterized by a self-perpetuating positive feedback loop with recruitment of various immune cells such as macrophages and polymorphonuclear leukocytes (PMNs) in all affected organs, keratinocyte proliferation and neutrophil accumulation in the skin, bone metabolism alterations in the joint and spine, and angiogenesis causing the clinical spectrum of PsA (63, 73, 74). Up until now, B cells are considered to have no profound role in this inflammatory response (75), but this is an ongoing debate in the field (71).

### Pivotal Cytokines Involved in the Immunopathogenesis of Psoriatic Arthritis

In the inflammatory response of PsA, various cytokines are released. Many of these cytokines are found in both PsO and PsA, but not all are equally present which may indicate at least partially different mechanisms of disease pathogenesis. One of the key cytokines is type I interferon (IFN), which is important for the onset of both the innate and adaptive immune response. Via release of IFNα by activated pDC in the skin (61), IFNα stimulates activation of myeloid DCs (57, 61, 65, 76) resulting in T-cell differentiation and proliferation as discussed earlier. IFNα activity is increased in psoriatic skin compared to unaffected skin (77) and in synovial fluid of patients with PsA compared to patients with osteoarthritis (60). Accordingly, activation of IFNα pathway in synovium is similar to skin (60) and suggests an important role for this cytokine at an early stage of the immunopathogenesis of PsA. Type II IFN, IFNγ, also induces different processes in PsA by activating antigen-presenting cells early in the psoriatic cascade (64). This cytokine is present in both PsA synovium and lesional skin, and levels correlate with disease severity (78). Blocking of IFNγ was earlier proposed as a potential treatment for PsA. However, so far no IFN blocking treatments have been developed for PsA, possibly because at the same time IL-17 targeting treatments emerged (64). However, IFN could still be of interest as is exemplified by the stimulation of keratinocytes in psoriasis-like skin in mice caused by IFNγ (79). This increase resulted in increased levels of Th1 and Th17 cells in the skin of these animals. Additionally, IFNγ can stimulate the inhibition or induction of osteoclasts depending on the relative levels of RANKL and IFNγ (36). The influence of IFNγ on both keratinocytes and osteoclasts suggests regulation of fundamental mechanisms by this cytokine. For this reason, further research into IFN for new treatment targets could be promising. One of the cytokines produced by activated myeloid DCs present in the skin is IL-23, which drives Th17- activation and differentiation. IL-23 is detected in psoriatic skin lesions and inflamed PsA synovium, though levels are highly variable between patients (63, 80). Blocking of IL-23 is very efficient for disease reduction in humans (81–83) and the pivotal role of IL-23 in PsA is also confirmed in K23 mice (35). In these mice, the induction of conditional transgenic expression of IL-23 in keratinocytes resulted in PsA-like symptoms with PsO like skin inflammation followed by arthritis, dactylitis and enthesitis. Th17 cells activated by IL-23 produce several cytokines including IL-17A, Il-17F, and IL-22. IL-17A is another essential cytokine in the pathogenesis and treatment of PsA (84). IL-17A can be produced by T helper cells as well as by innate (like) cells such as innate lymphoid cells (ILCs) (85), iNKT, γδT cells, MAIT cells. IL-17A blockade is effective in PsA (84, 86, 87) and results in a decrease in synovial macrophages and neutrophils as well as synovial IL-17A mRNA expression (88). In R26Stat3Cstopfl/fl CD4Cre mice in which the conditional allele of the hyperactive STAT3 gene, STAT3C, is expressed selectively in T lymphocytes resulting in enrichment of Th17 cells (29). It was shown that induced IL-17A expression resulted in both cutaneous and musculoskeletal PsA-like symptoms (Table 1). In addition, IL-17A minicircle (mc) injection induced systemic overexpression of IL-17A in C5BL/6J mice resulted in systemic bone erosions and PsO like skin lesions. When in these mice arthritis is induced by collagen (CIA model) then time to arthritis development is reduced with an increased arthritis severity (36). These findings also directly link IL-17A to skin and musculoskeletal symptoms resembling PsA (29). A cytokine that works synergistically with IL-17 is TNF, which is produced by many immune cells and is a key regulator of pro-inflammatory gene transcription, cytokine secretion, cytotoxicity and differentiation of T-helper cells (89). TNF is important for the induction of inflammatory responses, granulopoiesis, psoriasis skin lesions (90), enthesitis (52), and the pathological formation (91) and destruction of bone (92) via induction of RANKL (93). Blockade of TNF is very effective in PsA (94–96) and results in a decrease of inflammation and angiogenesis in skin (97, 98) and synovium (98, 99) and thus clinical PsA symptoms. Several other cytokines such as the IL-10 (100, 101) family [of which IL-22 (102, 103) more specifically], IL-17F (104), IL-36 (105–107), and IL-9 (108) are also suggested to play a role in the immunopathogenesis of PsA, but will not be further discussed here.

Even though the immunopathogenesis of PsA is incompletely understood, the above-mentioned studies indicate a role for both innate and adaptive immune cells, as well as for keratinocytes. The molecular mechanisms steering activation of those cells are yet to be further unraveled. However, it is clear that the IL-23/IL-17 axis and TNF are essential in establishing and enhancing the inflammatory response, resulting in the positive inflammatory feedback loop, bone formation and destruction. A limiting factor is that many of these human studies focused on patients with established PsA, while only a few studies have focused on early PsA patients. Furthermore, the PsA mice models do not always report the same clinical spectrum as human PsA. Therefore, more human studies and improved animal models are required focussing on the earliest or even preclinical phase of PsA.

## Challenges in Studying the Earliest Phases of Psoriatic Arthritis

Studies on early and preclinical disease are required as these studies will improve our knowledge on the pathogenesis of PsA and the transition of PsO to PsA which is essentially required if we ultimately want to come to preventive treatment or even a cure for PsA. Such studies will help to better understand the molecular pathways steering the transition from PsO to PsA and aid the identification of potential drug targets aimed at preventing the onset of PsA. Ultimately, this may lead to the development of risk stratification tools that identify those individuals at the highest risk of PsA who may benefit from preventative treatment. To study the earliest phases of PsA, consensus on the definition of early and preclinical PsA stages is needed as proposed in a recent review (22). A recent Delphi study could find consensus on some of the terminology, but especially the early phase needs further discussion (28). Such definitions will ease the use of preclinical terminology in studies that investigate clinical, imaging and molecular characteristics that determine the transition from PsO towards PsA in prospective cohort studies. These prospective studies will provide identification of novel risk factors associated with the development of PsA. So far, only a few prospective studies have been reported (109–111). An eight-year follow-up study in PsO patients confirmed nail involvement (RR 2.5, *p* = 0.002) associated with PsA development, additionally to a severe psoriasis phenotype (RR 5.4, *p* = 0.006), presence of low level of education (RR 0.30, *p* = 0.049), and uveitis (RR 31.5, *p* = 0.0002) (109). This study also showed that in PsO patients symptoms like arthralgia in women (HR 2.59, *p* = 0.02), fatigue (HR 2.36, *p* = 0.007), heel pain (HR 4.18, *p* = 0.02), and stiffness (HR 2.03, *p* = 0.045) at baseline were predictive for PsA development, as well as worsening of some of these symptoms (111). It is hypothesized that onset of clinically manifest PsA is preceded by a phase of subclinical enthesitis or arthritis (22). Subclinical inflammation could be visualized by various imaging techniques (22) and is not reflected by clinical symptoms. Subclinical enthesitis on ultrasound (US) was shown in the knees of around 83% of PsO patients compared to individuals without PsO that showed no signs of enthesitis on US (112). Another study using US in PsO patients showed subclinical enthesitis including grayscale and power Doppler in 49.3% in PsO patients compared to healthy controls (113). After 42 weeks of ustekinumab treatment in these PsO patients, their inflammation scores decreased by 47.5%. High-resolution peripheral quantitative CT in a prospective cohort of PsO patients with a mean follow-up of 28 months showed that subclinical enthesitis, characterized by structural entheseal lesions, was significantly associated with an increased risk for PsA development (HR 5.10, *p* = 0.008) (114). High volumetric cortical bone mineral density was associated with a lower risk for PsA development (HR 0.64). An imaging study with MRI in PsO patients has shown that synovitis in the hands of PsO patients combined with arthralgia, is suggestive of subclinical inflammation with a risk of 60% for PsA development after 1 year of follow-up (110). In a cohort of PsO patients with sub-clinical inflammation, characterized by synovitis and enthesitis on MRI and CT, this inflammation improved after 24 weeks of secukinumab treatment (115). Bone erosion and osteoproliferation remained stable during treatment and scores representing synovitis and tenosynovitis significantly decreased (*p* = 0.005). In this cohort also arthralgia significantly decreased (*p* = 0.003). Whether subclinical inflammation reflects an increased risk of development of clinically manifest disease needs further validation in additional prospective longitudinal studies. Prospective studies on both clinical and imaging factors in relation to disease development over time will help to identify specific groups that have a high risk for PsA development. In-depth tissue studies in this high-risk group to further understand the immunological alterations preceding disease onset may aid the development of innovative and even preventive therapies limiting disease progression.

## New Horizons for Translational Studies

As said, studying target tissues of early PsA patients and PsO patients with a high risk of PsA development will provide novel insights into the immunopathogenesis of PsA. Therefore, detailed analyses of tissues involved in the initial triggering of PsA are crucial. As earlier discussed, the onset of PsA is thought to take place outside the joints at sites such as entheses, skin and gut. Even though activated immune cells can migrate from affected psoriatic tissues to draining lymph nodes (LN) where they trigger T helper cell differentiation and initiate an inflammatory response, human lymphoid organs have not yet been studied in PsA. As LNs are the epicenter for T-cell activation and differentiated and activated T cells play a key role in PsA, it will be of great relevance to study those cellular responses in LNs, especially during the onset of PsA. Our research group has recently successfully initiated LN tissue sampling (116–119) of patients with inflammatory arthritis, RA-risk individuals and healthy controls (116–119). We have found altered frequencies of immune cells in LN biopsies of patients with RA and RA-risk individuals (117, 119). We could also show B cell depletion in these inguinal LN biopsies of RA patients after rituximab treatment, while switched memory B cells were more resistant to therapy (120). Preliminary findings in LN biopsies of patients with PsA revealed an unexpected increase in innate cells when compared with healthy LN tissue, which is under current investigation (data not shown). We postulate that LN tissue sampling and analysis will be highly valuable in further elucidating the immune responses involved in the pathogenesis of PsA in addition to studying all other involved tissues in PsA collected during the earliest or preclinical phase of disease. There have already been efforts made to investigate molecular differences between skin of PsO and PsA patients in cross-sectional studies. Differences have been reported (53, 54), but it is unclear if these observed molecular changes in skin precede or follow PsA development which stresses the importance to study patients during the transition from PsO to PsA using a prospective study design with serial tissue sampling. Although this is highly challenging, only this will show which molecular and cellular processes in the skin change during the transition from PsO to PsA. Additionally, this will show whether specific changes in the skin precede onset of musculoskeletal symptoms or whether the psoriatic skin changes after the onset of PsA as this is still under debate (121). Similar prospective follow-up studies could be applied to other target tissues, but tissues like bone and entheses are in general more challenging to collect in a prospective study. Studying synovial biopsies over time is challenging but possible, even in the absence of arthritis (122–128). When possible, it is preferred to study serial paired tissue samples of various tissues in the same patients. These studies are unfortunately sparse, which is not surprising, since the collection may be seen as too invasive for the patient. Due to the challenges of human in-depth tissue studies, animal models could support human studies. These animal models can give insights into immune alterations in various tissues which are difficult to study in humans, such as the entheses and bone. Moreover, by blocking specific pathways or molecules, causal relationships to development of disease can be investigated. But for this, improved animal models are needed that, contrary to the current models, better resemble the transition process from PsO to PsA in humans.

Concluding we have observed that many efforts have been made to unravel the immunopathogenesis of PsA, however prospective tissue studies in well-defined high-risk individuals are needed to further understand the disease pathogenesis and transition from PsO to PsA. Together with animal models that resemble this transition, these studies will provide further understanding of PsA development and ultimately may result in the development of treatments preventing the onset of PsA in PsO individuals at risk for this disease.

## Data Availability Statement

The original contributions presented in the study are included in the article/supplementary material, further inquiries can be directed to the corresponding authors.

## Author Contributions

All authors listed have made a substantial, direct and intellectual contribution to the work, and approved it for publication.

## Funding

LB received funding from a ZonMw VIDI project (91718371). MS received funding from a ZonMw VENI project (09150161810112). LB and MS received funding from the European Union's Horizon 2020 research and innovation programme under the Marie Skłodowska-Curie grant agreement No 847551 (ARCAID).

## Conflict of Interest

The authors declare that the research was conducted in the absence of any commercial or financial relationships that could be construed as a potential conflict of interest.

## Publisher's Note

All claims expressed in this article are solely those of the authors and do not necessarily represent those of their affiliated organizations, or those of the publisher, the editors and the reviewers. Any product that may be evaluated in this article, or claim that may be made by its manufacturer, is not guaranteed or endorsed by the publisher.
