# Corrigendum: Translational Research Studies Unraveling the Origins of Psoriatic Arthritis: Moving Beyond Skin and Joints

**DOI:** 10.3389/fmed.2021.761410

**Published:** 2021-09-08

**Authors:** Janne W. Bolt, Chaja M. J. van Ansenwoude, Ihsan Hammoura, Marleen G. van de Sande, Lisa G. M. van Baarsen

**Affiliations:** ^1^Department of Rheumatology & Clinical Immunology, Amsterdam Institute for Infection & Immunity, Amsterdam UMC, University of Amsterdam, Amsterdam, Netherlands; ^2^Department of Experimental Immunology, Amsterdam Institute for Infection & Immunity, Amsterdam UMC, University of Amsterdam, Amsterdam, Netherlands; ^3^Amsterdam Rheumatology & Immunology Center (ARC), Academic Medical Center, Amsterdam, Netherlands

**Keywords:** early psoriatic arthritis, psoriasis, immunopathogenesis, translational, animal models

In the original article, the **Funding** statement was inadvertently absent. The statement should be:

“LB received funding from a ZonMw VIDI project (91718371). MS received funding from a ZonMw VENI project (09150161810112). LB and MS received funding from the European Union's Horizon 2020 research and innovation programme under the Marie Skłodowska-Curie grant agreement No 847551 (ARCAID).”

The authors apologize for this error and state that this does not change the scientific conclusions of the article in any way. The original article has been updated.

## Publisher's Note

All claims expressed in this article are solely those of the authors and do not necessarily represent those of their affiliated organizations, or those of the publisher, the editors and the reviewers. Any product that may be evaluated in this article, or claim that may be made by its manufacturer, is not guaranteed or endorsed by the publisher.

